# Recurrence of macular corneal dystrophy on the graft 50 years after penetrating keratoplasty

**DOI:** 10.3205/oc000161

**Published:** 2020-08-06

**Authors:** Mona Bischoff-Jung, Elias Flockerzi, Andrea Hasenfus, Arne Viestenz, Pinio Matoula, Ursula Schlötzer-Schrehardt, Berthold Seitz

**Affiliations:** 1 Department of Ophthalmology, Saarland University Medical Center, Homburg, Germany; 2 Institute of Pathology, Saarland University Medical Center, Homburg, Germany; 3 Department of Ophthalmology, University of Erlangen-Nürnberg, Erlangen, Germany

**Keywords:** macular corneal dystrophy, recurrence, penetrating keratoplasty, hereditary corneal stromal dystroph

## Abstract

**Purpose:** To report the recurrence of a macular corneal stromal dystrophy 50 years after penetrating keratoplasty (PKP).

**Methods:** Observational case report

**Case** **description:** A 76-year-old male patient presented with visual impairment in the right eye (OD) 50 years after PKP in 1962 (44 years after PKP also in the left eye (OS) in 1968) following explosion injury. His visual acuity had already been impaired before the trauma because of bilateral corneal opacities. The central corneal thickness of the graft measured 584 µm (OD) and 544 µm (OS), whilst the peripheral host thickness (8 mm zone), however, was 1233 µm (OD, cranial) and 1131 µm (OS, nasal). The original graft diameter measured 6 mm in both eyes and the recipient cornea was cloudy and gray. The endothelial cell count was measured centrally (OD 1162 c/mm^2^, OS 1320 c/mm^2^). The visual acuity was 20/100 (OD) and 20/40 (OS). After excimerlaser-assisted repeated PKP (8.0/8.1 mm, OD), the histological analysis of the former graft revealed deposits of acid mucopolysaccharides (AMP) subepithelially, within the interface, in the donor stroma, and in the endothelium, which proved the peripheral recurrence of a macular corneal stromal dystrophy on the graft.

**Conclusion:** Recurrence of macular corneal stromal dystrophy is seldom, but it may occur many decades after PKP. In this patient, the host’s stroma was twice as thick as that of the graft. This may be caused by the active production of acid mucopolysaccharides in the host endothelium with secondary endothelial decompensation. Thus, PKP remains the gold standard in the cure of macular corneal dystrophy for long-term visual rehabilitation.

## Introduction

Macular corneal dystrophy is an autosomal-recessive inherited disease affecting the corneal stroma [1]. The deposition of acid mucopolysaccharides in Bowman’s layer, keratocytes and stroma, Descemet’s membrane and endothelium leads to the development of corneal opacities which also affect the corneal limbus. Manifesting in childhood, the cornea is usually thinner than normal but may develop an endothelial decompensation with consecutive thickening of the cornea during further progression of the disease [1]. Since no causal therapy exists up to now, most patients with corneal stromal dystrophies undergo penetrating keratoplasty (PKP) with the aim to restore visual acuity [2]. Acid mucopolysaccharide deposition may however recur in the corneal graft because of the persisting underlying disease, which is mainly attributed to the replacement of donor keratocytes by genetically defective host cells in macular corneal dystrophy [3]. Epithelial-stromal or stromal corneal dystrophies are reported to recur early on the graft after PKP: Granular corneal dystrophy is reported to show recurrence rates on the graft of about 40%, lattice corneal dystrophy of about 18% at 5 years follow-up after PKP [2]. Schnyder corneal dystrophy was reported to recur on the graft after 9 years [4]. There are different reports of recurrence time of macular corneal dystrophy on the graft after PKP, ranging from 20 months to 30 years [5]. Retrospective reports have shown that recurrences of macular corneal dystrophy developed more quickly in patients with smaller corneal graft diameters, and therefore it has been hypothesized that an increasing donor size would result in a longer graft survival rate [5]. The purpose of this report is to present a patient with acid mucopolysaccharide deposition in the periphery of a small corneal graft in the context of a repeated PKP for macular corneal dystrophy after 50 years.

## Case description

**Figure 1:**
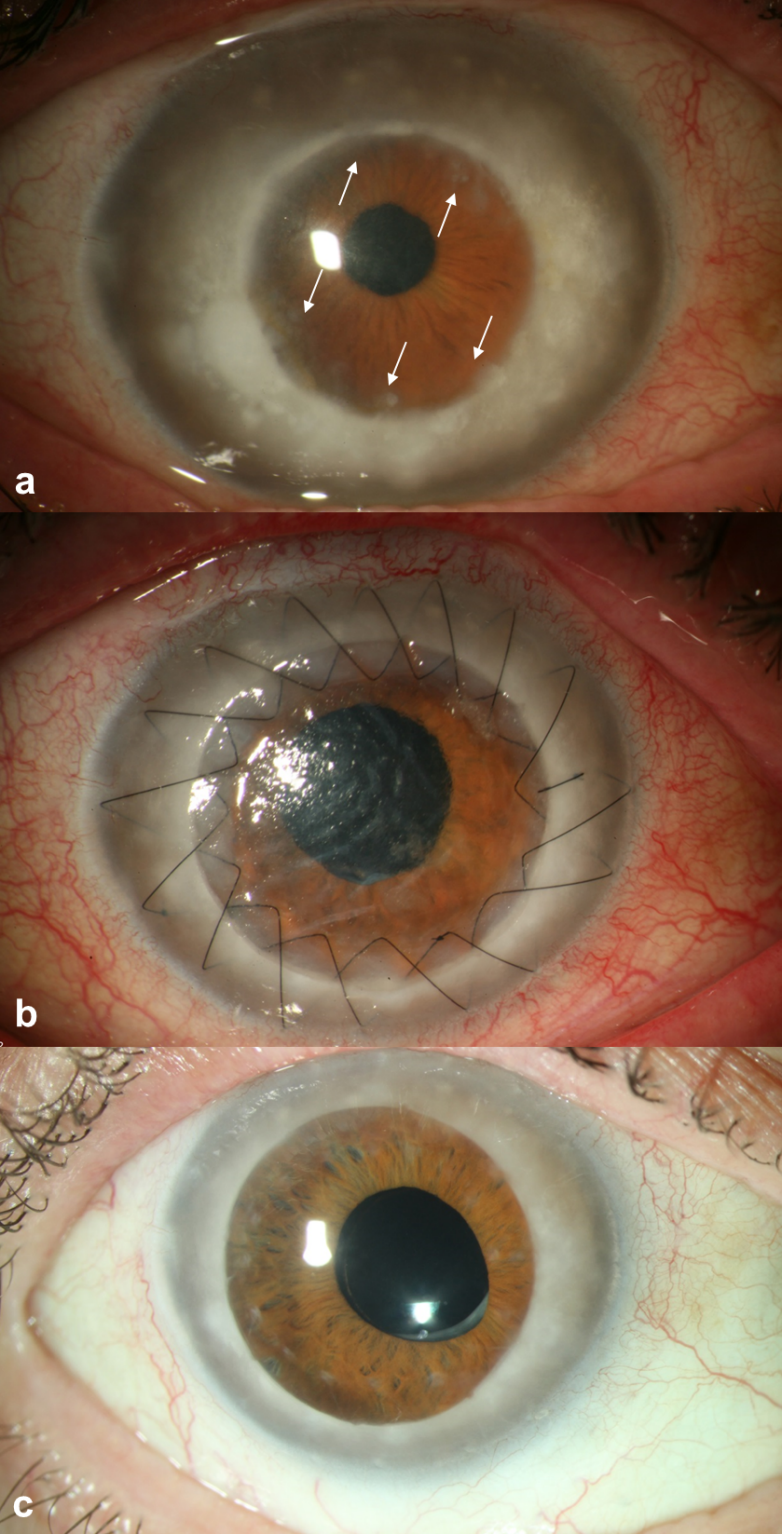
Slit lamp photographs a) Recurrence of macular corneal dystrophy on the corneal graft’s periphery (6 mm diameter, arrows); thickened host cornea with whitish deposits; b) Postoperative findings 4 days after surgery; graft with Descemet folds after excimerlaser-assisted repeated penetrating keratoplasty (8.0/8.1 mm diameter with double running cross-stitch suture according to Hoffmann); c) Clear corneal graft 40 months after repeated penetrating keratoplasty and 23 months after all-sutures-out; visual acuity 20/60

A 76-year-old male patient presented with visual impairment of the right eye 50 years after PKP (OD, 1962) and 44 years after PKP in the left eye (OS, 1968) following explosion injury. He reported that his visual acuity had already been impaired because of bilateral corneal opacities before the trauma in 1960. There were no corneal diseases in the family anamnesis. According to the patient, annual follow-up examinations were performed by his ophthalmologist during the 50 years after the first PKP. In addition, further controls were carried out in recent years due to primary open angle glaucoma. Cataract surgery was performed externally 42 years after PKP in the right eye. Six years after cataract surgery – in the meantime, a posterior capsulotomy had also been performed – the patient noticed a deterioration of his visual acuity in the right eye. In addition to the follow-up examinations of his ophthalmologist, the patient attended regular follow-up examinations for glaucoma in our department, where deposits in the periphery of the graft were described 48 years after the first PKP upon slit lamp examination. The graft diameter of the former grafts was 6 mm by slit lamp measurement. The central corneal thickness of the graft measured 584 µm (OD) and 544 µm (OS), whilst the peripheral host thickness (8 mm zone), however, was 1233 µm (OD, cranial) and 1131 µm (OS, nasal). The recipient cornea was cloudy and gray (Figure 1a (Fig. 1)). The endothelial cell count (measured centrally) was 1162 c/mm^2^ (OD) and 1320 c/mm^2^ (OS) with a best corrected visual acuity of 20/100 (OD) and 20/40 (OS). The patient was offered a repeated PKP on the worse right eye because of the progressive visual impairment and his discomfort caused by the blurred vision. The size of the new corneal graft was decided to be 8.0 mm, 2.0 mm larger than the former graft 50 years earlier. The cloudy appearance of the patient’s recipient cornea led to the suspicion of a macular corneal dystrophy. Surgery was performed without any complications (Figure 1b, c (Fig. 1)), and the excised primary corneal graft with a rim of the recipient cornea was sent to histopathological analysis. 

## Postoperative development and histological results

**Figure 2:**
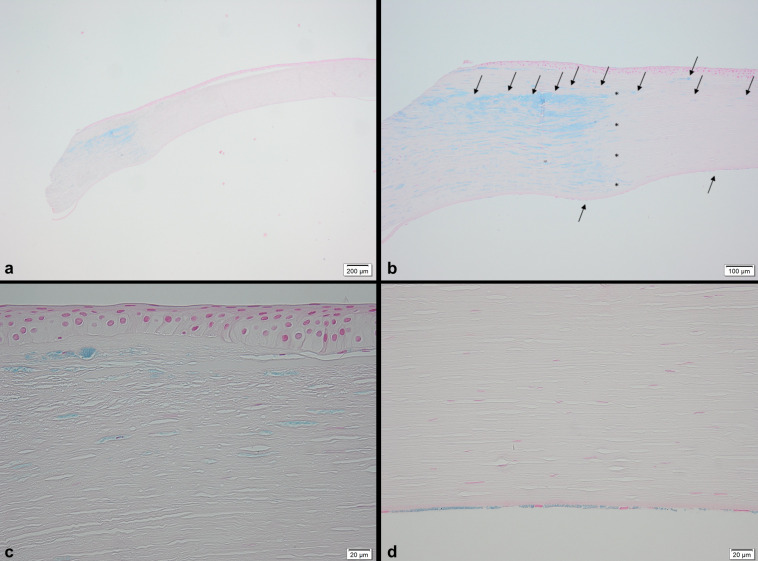
Histological analysis a) Overview: recipient cornea and part of the first graft, Astra 4x; b) Interface (asterisks): host cornea with deposits of acid mucopolysaccharides (blue, arrows) with transition to the interface zone and graft, Astra 10x; c), d) Central part of the first graft with deposits of acid mucopolysaccharides (blue) in the subepithelial stroma and in the endothelial cell layer, Astra 40x

**Figure 3:**
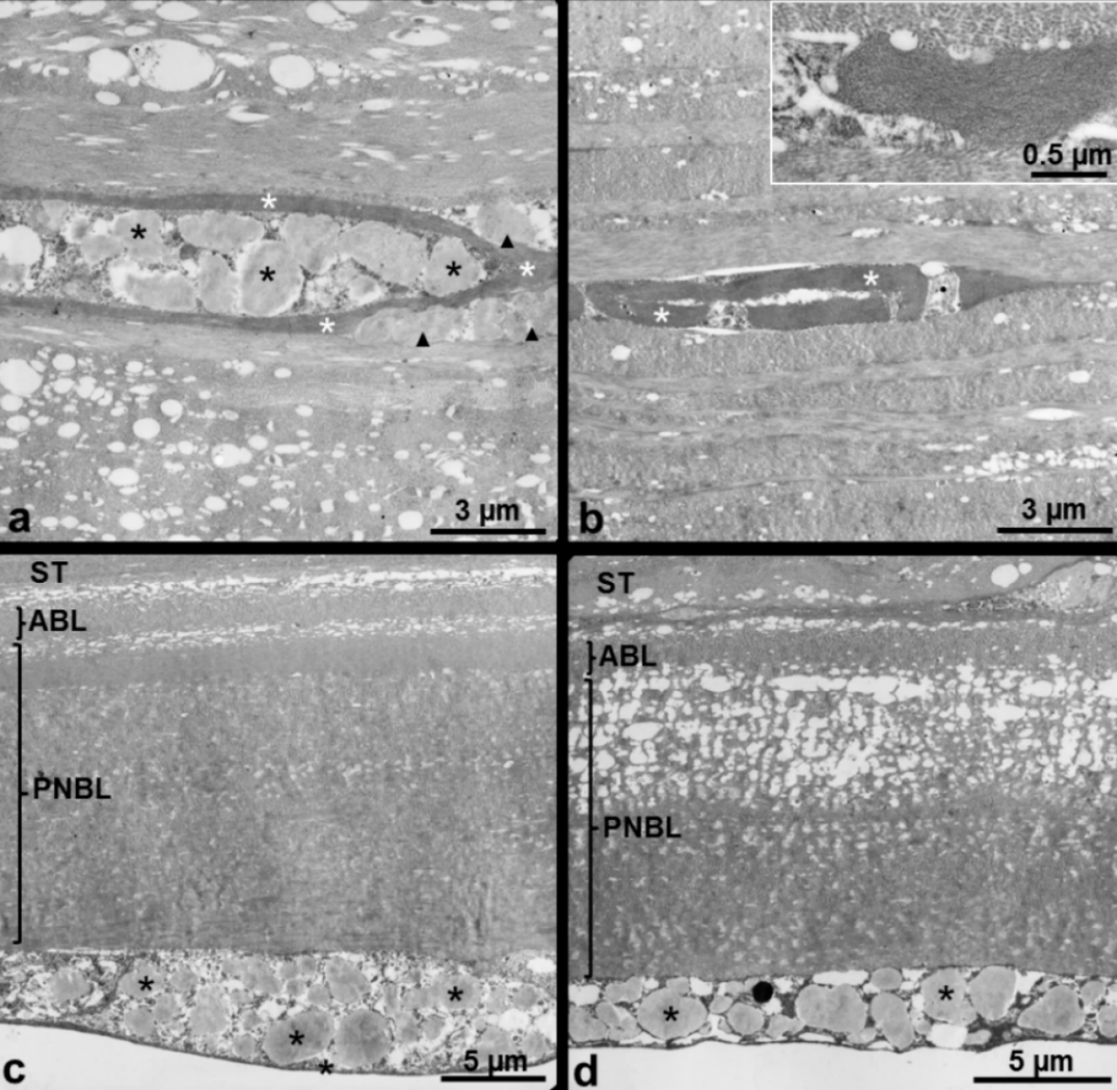
Transmission electron microscopy of the excised right cornea a) Donor corneal stroma of the peripheral part of the graft showing an extended keratocyte with intracellular (black asterisks) and extracellular (black arrowheads) deposits of a mucoid material together with extracellular deposits of a fibrillogranular material (white asterisks); b) Donor corneal stroma of the central part of the graft showing a keratocyte filled with fibrilllogranular material (asterisk); the insert shows the fibrillogranular material in higher magnification; c) Descemet’s membrane and endothelium of the donor cornea showing a normal anterior banded layer (ABL) and an abnormal posterior nonbanded layer (PNBL) of Descemet’s membrane as well as mucoid inclusions (asterisks) within endothelial cells; ST stroma; d) Descemet’s membrane and endothelium of the host cornea showing a normal anterior banded layer (ABL) and an abnormal posterior nonbanded layer (PNBL) of Descemet’s membrane as well as mucoid inclusions (asterisks) within endothelial cells; ST stroma

The histological analysis of the graft revealed deposits of acid mucopolysaccharides subepithelially, within the interface and the peripheral donor stroma, and in the endothelial cell layer (Figure 2a–d (Fig. 2)). These histological findings led to the diagnosis of a recurrence of macular corneal dystrophy within the 50-year-old corneal graft. The new graft showed an undisrupted postoperative healing (Figure 1b (Fig. 1)). In the long-term follow-up three years after repeated PKP, the best corrected visual acuity was 20/60 and there was no further recurrence of the macular corneal dystrophy (Figure 1c (Fig. 1)). Transmission electron microscopy revealed abundant intracellular and extracellular deposits of electron-lucent mucoid material and electron-dense fibrillogranular material within swollen keratocytes and the collagenous stroma of the graft (Figure 3a, b (Fig. 3)). Additionally, numerous electron-transparent lacunae and vacuoles were dispersed between the stromal collagen lamellae. These alterations were most pronounced in the peripheral portions of the donor cornea (Figure 3a (Fig. 3)), but could be also observed in the center of the graft (Figure 3b (Fig. 3)). Both donor (Figure 3c (Fig. 3)) and host endothelial cells (Figure 3d (Fig. 3)) contained inclusions of mucoid material indicating replacement of donor endothelium by diseased host endothelium. Whereas the anterior banded portion of Descemet’s membrane appeared unaffected in host and donor cornea, the posterior nonbanded portion was strikingly abnormal, showing vacuolar and fibrillary inclusions in both areas (Figure 3c, d (Fig. 3)).

## Discussion

The macular corneal dystrophy represents a classical stromal dystrophy, which is inherited autosomal-recessively and caused by a defect of the carbohydrate sulfotransferase 6 (CHST6) gene [1]. The disease is characterized by diffuse stromal haze and elevated irregular whitish deposits with no clear areas between. Affection of the endothelium may lead to corneal edema caused by endothelial decompensation [1]. This explains the thickening of the host cornea in our 76-year-old patient, which was nearly twice as thick as the first corneal graft in both eyes. Recurrences may occur many years after PKP due to the migration of keratocytes carrying the gene defect from the host to the donor cornea. Therefore, the graft’s periphery is first and most severely affected [5]. The smaller the graft, the higher is the risk for recurrence of macular corneal dystrophy on the graft for two reasons: First, more keratocytes carrying the gene defect are left in the host cornea; and second, these keratocytes may reach the center of the corneal graft faster by migration because of the shorter distance. Although data is rare, it seems that macular corneal dystrophy recurs earlier after lamellar keratoplasty (LKP) than after PKP. Robin et al. reported a patient showing recurrence 11 years after LKP [3]. Two other patients were reported who underwent repeated keratoplasty 18 and 19 years after LKP, respectively, because of recurrence of macular corneal dystrophy [6].

## Conclusions

Although appearing only after a long time, recurrence of macular corneal dystrophy is inevitable even decades after PKP due to the recurrent deposition of acid mucopolysaccharides within the corneal graft by keratocyte migration. PKP with a normal graft size of 8.0 mm seems to remain the gold standard in the cure of macular corneal dystrophy for long-term visual rehabilitation.

## Notes

### Competing interests

The authors declare that they have no competing interests.
